# Supplementary material for: Iron Uptake Controls Trypanosoma cruzi Metabolic Shift and Cell Proliferation
Source: Antioxidants (Basel). 2023 Apr 22;12(5):984. doi: 10.3390/antiox12050984 (PMC10215803; doi:10.3390/antiox12050984)
Supplement: Supplementary file 1 [file antioxidants-12-00984-s001.zip › antioxidants-2320474-supplementary.pdf]

Table S1. Primers informations.

Sequence name	Accesion number	E-value	r^2
T. cruzi iron transporter	TCDM_06386	94.26%	0.9948
T. cruzi ferric reductase	TCDM_07020	93.35%	0.9976
T. cruzi heme-regulated inhibitor	TCDM_00579	95.43%	0.9992
T. cruzi eukaryotic initiation factor 2α	TCDM_00323	90.04%	0.9816
T. cruzi protein kinase A	TCDM_05306	97.12%	0.9874
T. cruzi glyceraldehyde-3-phosphate dehydrogenase	TCDM_02134	93.63%	0.998
T. cruzi superoxide dismutase	TCDM_07362	92.93%	0.9987
T. cruzi ascorbate peroxidase	TCDM_04015	93.29%	0.9875
T. cruzi beta tubulin	BCY84_1376	93.73%	0.9816
